# Nutritional Status in University Students: Links to Physical Activity and Body Composition

**DOI:** 10.3390/nu18213577

**Published:** 2026-10-29

**Authors:** Manuela Filipec, Bojan Šarkanj, Natalija Uršulin-Trstenjak, Ivana Živoder, Nikolina Zaplatić Degač, Mihaela Kranjčević-Ščurić, Paulo Zekan

**Affiliations:** 1 Department of Physiotherapy, University North, 42000 Varaždin, Croatia; nursulin@unin.hr (N.U.-T.); izivoder@unin.hr (I.Ž.); nzaplatic@unin.hr (N.Z.D.); 2 Department of Food Technology, University North, 42000 Varaždin, Croatia; bsarkanj@unin.hr; 3 Department of Nursing, University North, 42000 Varaždin, Croatia; mkranjcevic@unin.hr; 4 Department of Obstetrics and Gynecology, University Hospital Sveti Duh, 10000 Zagreb, Croatia; paulo.zekan@gmail.com

**Keywords:** nutritional status, physical activity, body composition, university students

## Abstract

**Objective:** This study aimed to examine the associations between dietary consumption patterns, physical activity level, and body composition among students of biomedical sciences, and to determine whether dietary consumption patterns and body composition differed according to physical activity level. **Methods:** A cross-sectional study involved 187 ungraduated university students. The study was conducted among undergraduate physiotherapy students at University North, Varaždin, Croatia, with all measurements performed in the Physiotherapy Laboratory of the Department of Physiotherapy. Assessment of body composition was carried out using Body Composition Analyzer Tanita MC 780, nutritional status using the Food Frequency Questionnaire, and the physical activity using the International Physical Activity Questionnaire-long version. **Results:** The frequency of consumption of each category of food did not significantly differ between participants, with low, moderate and high levels of physical activity. Weak positive correlations were observed between the frequency of milk and milk product consumption and muscle mass (r = 0.153), and between sugar consumption and body fat (r = 0.189). No statistically significant differences were observed in dietary consumption patterns across physical activity levels or in body composition across physical activity groups. **Conclusions**: The findings provide descriptive information on dietary patterns, physical activity, and body composition among biomedical sciences students and may help identify areas for future health-promotion interventions and comparative studies in university populations.

## 1. Introduction

Physical inactivity and improper diet are one of the main risk factors for the development of chronic non-communicable diseases [1,2]. Improper eating habits such as eating fast food, eating high-calorie foods, foods poor in nutrients, skipping and missing meals cause health problems and nutritional deficits. Research shows a significant decline in physical activity and an increase in a sedentary lifestyle, especially in the period of adolescence and young adulthood [2,3]. Sedentary behavior is associated with numerous adverse physiological health outcomes: morbidity and mortality [4].

Physical activity is an important factor that contributes to energy consumption and is related to nutritional status, which is described as the energy balance of nutritional intake with the energy needs of the body. In recent years, obesity in early adulthood has become a worrying global public health problem [5,6]. Research shows that people with reduced physical activity have a 1.325 times greater risk of obesity than those who engage in moderate and high physical activity [7].

Nutritional status is determined by nutritional nutrients in an individual’s diet and represents a significant component of health [7,8]. Also, intake of necessary nutrients, physical activity and bone density are closely related to predictors of general health and point to the need for their monitoring at the earliest possible age [5,6].

It is believed that the adjustment period at the university affects the maintenance of proper eating habits and physical activity, i.e., it is associated with weight gain, unhealthy eating, sleep problems and lack of physical activity. These problems are especially present in the first year of studies, that is, at the beginning of university education level [9].

The main obstacles identified among students related to physical activity are lack of time, motivation and available places. Although they believe that exercise is a vital factor in an adult’s life, students claim that they lack time but still do not consider themselves to lead a sedentary lifestyle [3,4].

Understanding the variable factors that improve and maximize bone mass at a young age is necessary for creating feasible programs for the prevention of osteoporosis as one of the significant problems of old age [8]. Adequate nutrition is critical in the student population for psychophysical health, cognitive and intellectual development, and achieving optimal academic success. Therefore, the dietary and physical activity patterns of college students have attracted worldwide attention in recent years. For example, the identification of predictors of bone status indicates that muscles and bones are interrelated with physical activity and fitness in young adults [8]. Calcium and vitamin D intake are key to maintaining bone health [10], but there are also additional nutritional substances such as magnesium, vitamin C and vitamin K that affect health and disease prevention [11,12,13].

The nutritional behavior of students and food intake is influenced by a number of factors related to personal knowledge and skills, social factors, environmental factors, and factors of the educational institution itself [14]. It has been observed that students with better knowledge about nutrition consume less unhealthy fats and cholesterol, which increases the importance of education on nutrition as a potential tool in health campaigns to promote a proper diet among students [15]. At the same time, students with a higher level of physical activity consume smaller amounts of simple carbohydrates (galactose and sucrose) compared to students with less physical activity [16].

A recent study among female university students demonstrated that physical activity level was positively associated with skeletal muscle mass index and negatively associated with body fat percentage, indicating that lower physical activity may be accompanied by an unfavorable body composition profile [17]. Furthermore, recent evidence suggests that conventional indicators of nutritional status, such as body mass index (BMI), may not fully reflect differences in body composition. For example, a 2025 study of 993 university students identified hidden obesity, characterized by excessive body fat despite a normal BMI, in 17% of participants [18]. Similarly, research among university students has demonstrated that periods characterized by reduced physical activity and poorer dietary habits may coincide with increased fat mass, decreased muscle mass, and increased visceral fat [19]. Other recent studies have also emphasized relationships among eating behavior’s, nutritional status, physical activity, and different components of body composition in university populations [20,21]. Despite numerous studies on the physical activity and eating habits of students, there is insufficient data on the connection between physical activity and the nutritional status of students. Therefore, this study aimed to examine the associations between dietary consumption patterns, physical activity level, and body composition among students of biomedical sciences, and to determine whether dietary consumption patterns and body composition differed according to physical activity level.

## 2. Materials and Methods

### 2.1. Study Design and Participants

This is a cross-sectional study conducted among undergraduate physiotherapy students at University North, Varaždin, Croatia, between March and June 2024. All procedures employed in this study were conducted in accordance with the ethical principles of the Helsinki Declaration and were approved by the University North Ethics Committee (approval number: 641-01/24-01/04, 26 February 2024). Participants were provided with comprehensive information about the procedures to be performed and were asked to sign informed consent forms. All participants provided written informed consent. All measurements were conducted in the Physiotherapy Laboratory of the Department of Physiotherapy. A convenience sampling approach was used, and all eligible undergraduate physiotherapy students were invited to participate. No a priori sample-size calculation was performed, as the study was designed as an exploratory cross-sectional investigation. Students from the first, second, and third years of the undergraduate physiotherapy program were invited to participate. Recruitment was conducted through invitations provided during lectures and an announcement distributed via the university’s Merlin student learning platform. Participation was voluntary, and students who met the predefined eligibility criteria and provided informed consent were included in the study. A total of 288 students were eligible for participation. Of these, 187 students participated in the study, corresponding to a participation rate of 64.9%.

Inclusion criteria were age between 18 and 25 years and absence of chronic systemic diseases requiring medication. Exclusion criteria were pregnancy, recent surgery, rheumatological, neurological and musculoskeletal diseases, gastrointestinal diseases, and current use of medications influencing metabolism or body composition. Of the 194 participants, 7 were excluded from the study because they did not meet the inclusion criteria (they were older than 25 years).

### 2.2. Measurements

The data were collected using a quantitative method, including the results of questionnaires and measurements from the body composition analyzer. Assessment of nutritional status was carried out using the Food Frequency Questionnaire (FFQ), assessment of the level of physical activity was carried out using the International Physical Activity Questionnaire-long version (IPAQ-L) and body composition parameters were obtained using Body Composition Analyzer Tanita MC 780. Demographic data, including age and gender, were also collected.

### 2.3. Assessment of Nutritional Status

The Food Frequency Questionnaire (FFQ) is a widely used dietary assessment tool that includes a list of foods and beverages, portion sizes, and consumption frequencies [22]. Dietary habits were assessed using an adapted version of the FFQ developed and validated for the Croatian adult population by Babić et al. [23]. The original questionnaire was modified and expanded to include 184 food items to better reflect the dietary habits of the study population. The FFQ used in this study consists of 184 items, divided into nine domains: milk and milk products; meat, poultry, fish, eggs and products; fats, oils and products; legumes, cereals and products; sugar, honey and sweets; vegetables and products; fruit and products; drinks and beverages; snacks and cakes. Participants choose the frequency of consumption (rarely, occasionally, frequently) of the foods and beverages in the past month. For the purpose of the present analysis, a study-specific scoring procedure was used to quantify the reported frequency of consumption within each food category. Responses were classified into three categories: rare consumption (2–3 times per month), occasional consumption (1–2 times per week), and frequent consumption (4–6 times per week). Each item was assigned 0 points for rare consumption, 1 point for occasional consumption, and 2 points for frequent consumption. The sum of the item scores was used as an indicator of the reported consumption frequency for each food category.

### 2.4. Assessment of Physical Activity

The IPAQ-L is a 27-item self-reported measure of physical activity through five categories: job-related physical activity; transportation physical activity; housework, house maintenance, and caring for family; recreation, sport, and leisure-time physical activity; time spent sitting [24]. Based on the scoring protocol, participants were classified into three physical activity levels: low, moderate, and high. All continuous scores are expressed in MET-minutes/week and scored according to the Guidelines for Data Processing and Analysis of the International Physical Activity Questionnaire [25].

### 2.5. Body Composition Measurements

Body composition was measured using a Body Composition Analyzer MC-780 (Tanita Europe, Amsterdam, The Netherlands). Measurements were conducted in the morning following an overnight fast to ensure accuracy and reliability. Participants wore a single layer of clothing, without shoes or socks. Measurement were performed in standing position with both feet parallel on the electrodes without bending knees. Participants were instructed to grasp the handle, placing their thumbs on the oval-shaped electrodes. Additionally, they were guided to keep their arms extended and avoid contact with other parts of their body. The entire procedure took approximately one minute. The following parameters were assessed: Body mass index (BMI), Body fat mass (FATM), Body fat percentage (FATP), Visceral fat level (VFATL), Fat-free mass (FFM), Muscle mass, Bone mass, Total body water (TBW), Intracellular water (ICW), Extracellular water (ECW), Basal metabolic rate (BMR), Physique rating and Metabolic age.

Height was measured using a SECA stadiometer (SECA GmbH & Co. KG, Hamburg, Germany), with participants standing fully erect, feet together, head positioned in the Frankfort horizontal plane, and arms hanging freely. Body weight was measured using a Tanita MC-780, with participants standing barefoot and motionless on the analyzer platform. The measured height was entered into the Tanita MC-780, which automatically calculated BMI based on body weight and height and reported the value in kg/m^2^.

### 2.6. Statistical Analyses

For statistical analysis, the software Jamovi was used (The Jamovi Project, 2024. Jamovi (Version 2.3.16.0) [Computer Software]. Retrieved from https://www.jamovi.org, Sydney, Australia). The normality of data distribution was assessed using the Shapiro–Wilk test. The analysis indicated that the data were not normally distributed; therefore, non-parametric statistical methods were used for the analysis. Data are expressed as median with IQR for continuous variables and absolute and relative frequencies for categorical variables. A Kruskal–Wallis non-parametric test was used to investigate whether there was difference in consumption frequency between different levels of physical activity for each category of food. Spearman correlation was used to investigate association between consumption frequency and body composition for each category of food. In order to investigate effect of physical activity level on body composition Kruskal–Wallis non-parametric test was used. Statistical significance was set at *p* < 0.05.

## 3. Results

A total of 187 participants were included in the study, including 139 (74.3%) female and 48 (25.7%) male. The median age of the participants was 20 years (IQR, 20–21), the median BMI was 23.1 kg/m^2^ (IQR, 20.8–26.1), the median height was 168 cm (IQR, 164–176), and the median weight was 65 kg (IQR, 57.1–76.95).

Participants reported the highest median duration of physical activity in the domain of housework, house maintenance, and caring for family (47 min/day), followed by recreation, sport, and leisure-time activities (35 min/day). The median sitting time was 300 min/day. According to the IPAQ classification, 40.6% of participants had low, 36.9% moderate, and 22.5% high physical activity levels. Detailed results of the body composition parameters and physical activity assessed using the IPAQ are presented in Table 1.

Regarding dietary patterns, the most frequently consumed foods within each food group were cow’s milk (55%), yogurt (11%), and Gouda cheese (8%) among milk and dairy products; chicken breast (24%), eggs (20%), and pork (7%) among meat, poultry, fish, and eggs; sunflower oil (29%), olive oil (12%), and pumpkin oil (12%) among fats and oils; wheat bread (18%), mixed bread (11%), and oat flakes (10%) among legumes and cereals; sugar (36%), milk chocolate (10%), and honey (5%) among sugar, honey, and sweets; lettuce (24%), garlic (16%), and carrots (14%) among vegetables; bananas (21%), apples (10%), and blueberries (3%) among fruits; cakes (3%), chips (2%), and sticks (2%) among snacks and cakes; and water (64%), coffee (24%), and fruit juice (16%) among beverages.

Results show that the frequency of consumption of each category of food did not significantly differ between participants with low, moderate and high-level of physical activity (Table 2).

Muscle mass showed weak but significantly positively correlated with frequency of milk and milk product’s consumption (ρ = 0.153), while body fat positively correlated with frequency of sugar consumption (ρ = 0.207). Furthermore, meat consumption positively correlated with muscle mass (ρ = 0.158) and drink consumption positively correlated with total body water (ρ = 0.152). On the other hand, all other body composition parameters did not correlate with frequency of consumption for each food category (Table 3).

Results show that the level of physical activity did not influence any body composition parameter (Table 4).

## 4. Discussion

The present study examined dietary consumption patterns, physical activity levels, and body-composition characteristics among undergraduate physiotherapy students.

The predominance of female participants reflects the composition of the present sample and should be considered when interpreting the findings. Gender distribution may differ substantially between academic programs and institutions; therefore, the present sample cannot be assumed to represent the gender distribution of university students generally. The median age of 20 years indicates that the participants were young adults, a period during which dietary and physical activity behaviors may be established and maintained into later adulthood. Previous research has emphasized the importance of this life stage for the development of health-related behaviors [26]. According to the World Health Organization (WHO) [26] adolescence and early adulthood represent a “critical window” for the adoption of healthy behavioral patterns that significantly affect the risk of chronic disease later in life. Nevertheless, the cross-sectional nature of the present study does not allow conclusions regarding changes in these behaviors over time.

Participants demonstrated BMI values within the normal-weight range according to the WHO classification [27]. However, BMI should be interpreted alongside other body-composition measures and lifestyle characteristics rather than as a standalone indicator of nutritional status. Although participants were distributed across low, moderate, and high physical activity categories according to the IPAQ-L, all reported some level of physical activity, and no completely inactive comparison group was available in our study. This may have limited variability between activity groups and partly explain the absence of statistically significant differences in body-composition parameters.

On the other hand, the median sitting time was 300 min per day, indicating a considerable amount of sedentary behavior. Prolonged sitting is common among university students and may occur in the context of lectures, studying, computer use, and other academic activities. However, sitting time and physical activity represent related but distinct behavioral dimensions; participation in physical activity does not necessarily eliminate prolonged sedentary time. The current WHO recommendation does not specify a quantitative daily threshold for sedentary time in adults but recommend limiting the amount of time spent sedentary and replacing sedentary time with physical activity of any intensity [26]. Previous research has linked sedentary behavior among university students with adverse physiological health indicators [28]. Therefore, the relatively high sitting time observed in the present sample provides an additional aspect of student lifestyle that should be considered alongside physical activity level rather than being used to characterize participants as either active or inactive.

Our results did not show statistically significant differences in body composition parameters among participants with different levels of physical activity intensity. This finding is consistent with the study by Alsubhi and Turkistani [29], who reported no significant differences in body composition between physically active and inactive female university students. However, it contrasts with the findings of Oshita et al. [17], who observed significant associations between physical activity level, skeletal muscle mass index, and body fat percentage among Japanese female university students. These contrasting findings suggest that the relationship between physical activity and body composition may vary according to the characteristics of the study population, the distribution of physical activity levels, and the indicators used to assess body composition. Several methodological and population-related factors may help explain the absence of significant differences in our study. First, all participants reported at least some physical activity, and therefore, a completely inactive comparison group was not available. This may have restricted the range of physical activity exposure and reduced the likelihood of detecting between-group differences. Second, the IPAQ-L provides a self-reported estimate of physical activity but does not fully distinguish between exercise modalities, particularly resistance training, which is relevant to muscle mass development. Third, body composition is influenced by several interacting factors, including dietary intake, energy balance, sedentary behavior, and individual characteristics. Consequently, differences in physical activity categories alone may not fully explain variability in body composition.

In our study, a weak positive association was observed between the frequency of dairy product consumption and muscle mass. Given the small magnitude of this association and the cross-sectional study design, this finding should be interpreted cautiously and cannot be considered evidence that dairy consumption contributes to greater muscle mass in this population. Although casein and whey proteins have been associated with stimulation of protein synthesis and the maintenance and enhancement of lean body mass [30,31], these established effects do not establish a causal relationship between dairy consumption and muscle mass in the present study. Furthermore, similar associations have been reported in studies of young adults, with regular consumption of dairy products, such as yogurt, cheese, and milk, being associated with greater muscle mass [32,33]. This association may be related not only to the protein content of dairy products, but also to the presence of calcium, vitamin D, and other nutrients that support muscle function and metabolism. The practicality and availability of dairy products may make them a convenient dietary choice for students and may be associated with broader dietary and lifestyle patterns [34]. Furthermore, research examining body composition in university students has highlighted the potential relevance of multiple lifestyle factors. For example, Oshita et al. [17] found that skeletal muscle mass index was associated with physical activity and chronotype, emphasizing that muscle mass should not be interpreted in relation to a single food group. In our study, dairy consumption was assessed using reported consumption frequency rather than total protein intake or total energy intake. Consequently, we could not determine whether participants who consumed dairy products more frequently also had higher overall protein intake or different dietary patterns. Taken together, the weak correlation observed in our study should be considered exploratory and requires confirmation in studies using quantitative dietary assessment and appropriate adjustment for potential confounding factors.

Similarly, the association between the frequency of consumption of sugar, honey, and sweets and body fat was weak. This finding should be interpreted in the context of a physically active sample and in light of existing research linking higher intake of refined sugars to increased visceral adiposity and insulin resistance [35]. However, evidence concerning sugar-sweetened beverages cannot be directly extrapolated to all sugar-containing foods, including honey and sweets, because their nutritional composition and patterns of consumption differ. Although the consumption of sugar-rich foods may contribute to excessive energy intake when it is not balanced by energy expenditure, the frequency of consumption alone does not establish the amount consumed or its contribution to total energy intake. Research among university students has also demonstrated associations between dietary habits and physical activity, suggesting that dietary behaviors should be interpreted within the broader lifestyle context [36]. Furthermore, differences in portion sizes, food composition, and overall energy balance may partly explain why the frequency of consumption of a particular food group does not necessarily correspond to a strong association with body composition. Given the cross-sectional design and the lack of comprehensive adjustment for potential confounders, this weak correlation should be interpreted cautiously as an exploratory finding rather than evidence of a causal relationship between the consumption of sugar, honey, and sweets and body fat.

Furthermore, given the generally weak associations and the multiple statistical comparisons performed, the possibility of false-positive findings cannot be excluded. Therefore, these findings should be interpreted cautiously and considered exploratory, requiring confirmation in future studies with larger samples and appropriate adjustment for multiple comparisons and potential confounding factors.

Beyond the observed associations between individual dietary components and body composition, the reported frequency of food consumption provides insight into the broader dietary pattern of the study population. Milk and milk products, meat and eggs, and several fruit and vegetable items were among the frequently reported foods, while legumes and cereals were less prominent. Water was the most frequently reported beverage, whereas snacks and cakes were reported less frequently. This dietary pattern diverges from findings in previous studies on student populations, where refined grains and nutrient-poor carbohydrates are the predominant choices [37,38]. Furthermore, these findings reflect a favorable hydration pattern and low intake of energy-dense, nutrient-poor foods, representing a positive deviation from earlier data showing frequent consumption of sugary drinks and snacks among university students [39]. Notably, chicken breast and eggs were among the most commonly consumed foods. Both are nutrient-rich foods and important sources of high-quality protein, which may be relevant to individuals who engage in regular physical activity [40]. This interpretation should be considered in the context of the present sample, in which all participants reported some level of physical activity. However, when compared with current dietary guidelines, the reported intake of fruits and vegetables appears to fall short of the recommended minimum of 400 g per day [41]. Similar dietary inadequacies among university students have been reported by Wirnitzer et al. [42], including suboptimal consumption of fruits, vegetables, and whole grains. Although fruits, vegetables, legumes, and whole grains were reported among the consumed food groups in the present study, their frequency of consumption suggests that there is still scope for improving dietary diversity. WHO recommendations emphasize the importance of a diet rich in fruits, vegetables, whole grains, and other nutrient-dense foods [41], suggesting that awareness and knowledge of healthy nutrition do not necessarily translate into consistent healthy dietary practices [43]. Consequently, the observed frequencies cannot be used to determine whether participants met recommended intakes for specific food groups or nutrients, nor do they allow firm conclusions regarding overall diet quality. These findings should therefore be interpreted primarily as descriptive indicators of food-choice patterns within this sample. The dietary findings should also be considered in relation to recent research on university students’ health-related behaviors. Lonati et al. found a positive association between physical activity and healthier dietary behaviors, although suboptimal lifestyle behaviors were still reported within the student population [44]. These findings suggest that health-related behaviors may cluster together but are not necessarily uniformly favorable in all students. In the present study, the relatively frequent consumption of some protein-rich foods and water may indicate potentially favorable individual food choices; however, the reported frequency of food consumption does not provide sufficient information to establish overall dietary quality or adherence to dietary recommendations. Similarly, the lower reported frequency of some food groups cannot be interpreted as evidence of inadequate nutrient intake without quantitative assessment. These findings reinforce the importance of distinguishing individual food-consumption frequencies from overall dietary patterns and nutritional adequacy. Differences between studies may also reflect variation in food-frequency assessment methods, cultural dietary patterns, access to food, and the university environment.

Sex-related differences should also be considered when interpreting the associations between dietary habits and body composition observed in the present study. Men and women differ in body composition, particularly in skeletal muscle mass, total body fat and regional fat distribution, partly due to differences in sex hormone regulation and metabolic physiology [45]. Moreover, studies involving university students have reported sex-related differences in dietary habits and body composition [18,46]. These findings suggest that the associations between dietary habits and body composition may differ between male and female students. Therefore, the pooled analysis in the present study may have obscured potential sex-specific patterns or resulted in associations that were more representative of the predominantly female sample. However, as sex-stratified analyses were not performed, the extent to which these differences influenced the observed findings remains uncertain.

Taken together, the findings indicate that the dietary behaviors observed in this sample cannot be readily characterized as either uniformly favorable or unfavorable. Although some potentially favorable food choices were frequently reported, the frequency-based dietary assessment does not allow conclusions regarding overall diet quality or adherence to quantitative dietary recommendations. These findings highlight the potential value of targeted preventive interventions within educational institutions. Programs integrating nutrition education, increased availability of healthy food options, and promotion of physical activity have been reported to improve health-related outcomes among student populations, including over relatively short intervention periods [47]. Furthermore, the present study did not assess the broader university environment, including the availability, accessibility, and affordability of healthy food options, opportunities and facilities for physical activity, or institutional health-promotion policies. Consequently, an important contextual factor when interpreting the present findings is the nutritional and physical activity environment at University North. The university currently does not have a student canteen at University North. Therefore, students do not have access to meals provided directly by the university during the study day and may obtain food primarily from grocery stores and bakeries in the surrounding area. This context may influence students’ food choices and should be considered when interpreting the dietary patterns observed in the present sample. In contrast, opportunities for organized physical activity are available within the university environment. University North supports student participation in sports, while organized student sport is provided through Unisport UNIN, which offers opportunities for training and participation in sports competitions. These opportunities may facilitate engagement in physical activity alongside students’ academic responsibilities. Nevertheless, the present study did not assess students’ participation in these sports programs or their use of food outlets in the surrounding area. Therefore, it is not possible to determine whether the institutional environment directly contributed to the dietary and physical activity patterns observed in our sample. Future studies should therefore examine university-level environmental and policy factors alongside individual dietary and physical activity behaviors to better understand the contextual determinants of students’ lifestyle patterns.

Several limitations should be considered when interpreting the findings. First, this exploratory cross-sectional study was based on a convenience sample, and no a priori sample-size calculation was performed. The sampling approach limits the generalizability of the findings, while the cross-sectional design precludes conclusions regarding causality or changes in dietary habits, physical activity, or body composition over time. Furthermore, the absence of an a priori sample-size calculation and the relatively small number of participants in some physical activity categories may have limited statistical precision and the ability to detect small associations or between-group differences. Second, although the FFQ was adapted from a previously validated questionnaire, the modified version and the study-specific scoring procedure were not independently validated. These modifications may have affected the measurement properties of the original instrument, while the categorization of consumption frequencies into three levels may have reduced sensitivity to differences in dietary habits. Therefore, the findings should be interpreted cautiously, as they reflect self-reported consumption frequencies rather than precise estimates of dietary intake. Third, the sample consisted exclusively of physiotherapy students enrolled at University North, with a predominance of female participants, which limits the generalizability of the findings to students from other academic disciplines, universities, or demographic groups. Therefore, future studies involving more diverse student populations are needed to establish the applicability of these findings to broader student populations. An additional limitation of this study is that the results were not analyzed separately by sex. Although both male and female students were included, the study was primarily designed to address its main research objectives rather than to investigate sex-specific differences. The sample was predominantly female, and the unequal distribution of male and female participants may have limited the precision of comparisons between the two groups. As sex-stratified analyses were not performed, we could not determine whether the observed associations were consistent across sexes or whether the overall findings were predominantly influenced by the larger subgroup of female participants. Future studies with more balanced sex distributions and appropriately powered sex-stratified analyses are warranted to investigate these potential differences. Fourth, all participants reported at least some level of physical activity; therefore, the study did not include a truly inactive comparison group. Fourth, the absence of a comparison group from non-biomedical academic programs limits the ability to determine whether the observed dietary and body-composition characteristics are specific to students with a health-related educational background. Fifth, potential confounding variables such as socioeconomic characteristics, total energy intake, or other lifestyle behaviors were not controlled for in the statistical analyses. Therefore, the observed associations should be interpreted with caution, as the influence of unmeasured or uncontrolled confounding factors cannot be excluded. Finally, institutional factors, such as the availability of food services, opportunities for organized physical activity, and university health-promotion policies, may influence students’ dietary and physical activity behaviors. However, these factors were not directly assessed in the present study and should therefore be considered when interpreting and comparing the findings with those from universities with different institutional environments.

Despite these limitations, the scientific contribution of the present study lies in its integrated assessment of dietary habits, physical activity, and body composition among undergraduate physiotherapy students, a population of future healthcare professionals. Importantly, the findings provide additional insights into the complexity of these relationships, showing that differences in self-reported physical activity levels were not accompanied by statistically significant differences in body composition, while associations between the frequency of consumption of specific food groups and body composition parameters were generally weak. These findings highlight the importance of considering dietary habits, physical activity, and body composition as interrelated but distinct aspects of health-related behaviors, rather than assuming that higher physical activity levels or more frequent consumption of particular food groups necessarily correspond to more favorable body composition. Furthermore, the findings provide a foundation for future research employing more comprehensive dietary assessments and objective measures of physical activity to better understand these relationships in university student populations.

## 5. Conclusions

The study highlights that undergraduate physiotherapy students at University North generally demonstrated favorable BMI and body composition characteristics and reported engagement in physical activity, although prolonged sedentary behavior and suboptimal consumption of several food groups were also observed. These findings provide a descriptive profile of this specific student population and may help identify areas for further investigation rather than indicating an immediate need for a specific preventive program. Future research should examine the extent to which university policies, infrastructure, and opportunities for organized physical activity may influence students’ dietary and physical activity behaviors. Longitudinal and intervention-based studies involving students from different academic disciplines and universities are also needed to determine whether targeted approaches can improve dietary behaviors, reduce sedentary time, and contribute to healthier body-composition profiles.

## Figures and Tables

**Table 1:** Results of IPAQ questionnaire and body composition.

Domain	MET	Duration	Frequency
	(Min/Week)	(Minutes Per Day)	(Days)
Job-related	0.00 (0.00–4438)	0 (0–153)	2 (0–7)
Transportation	231 (0.00–714)	10 (0–30)	3 (0–6)
Housework, house maintenance, caring for family	1188 (458–2796)	47 (17–96)	5 (3–7)
Recreation, sport, and leisure-time	1023 (248–2772)	35 (10–86)	5 (2–7)
Time spent sitting	0.00 (0.00–0.00)	300 (180–360)	
Total MET	5613 (2519–10140)		
PHYSICAL ACTIVITY LEVEL	**N (%)**		
Low	76 (40.6%)		
Moderate	69 (36.9%)		
High	42 (22.5%)		
BODY COMPOSITION	**Median with IQR**		
Body fat	0.41 (0.28–0.54)		
FATP	22.1 (16.8–28.0)		
FATM	14.1 (10.1–19.6)		
FFM	49.1 (44.8–58.3)		
VFATL	1.00 (1.00–3.00)		
Muscle mass	0.41 (0.33–0.45)		
Bone mass	2.50 (2.30–3.10)		
TBW	33.1 (30.3–41.3)		
ECW	14.7 (13.1–17.6)		
ICW	18.2 (17.1–23.5)		
BMR	6330 (5740–7440)		
Metabolic age	16.0 (12.0–26.0)		
Physique Rating	44.0 (35.0–53.5)		

Data are expressed as median with IQR for continuous variables and absolute and relative frequencies for categorical variables. FATP (%)-Body fat percentage, FATM (kg)—Body fat mass, FFM (kg)—Fat-free mass, VFATL (unitless level), Visceral fat level, TBW (kg)—Total body water, ECW (kg)—Extracellular water, ICW (kg)—Intracellular water (ICW), BMR (kJ/day)—Basal metabolic rate. The Visceral Fat Rating was classified according to the reference scale automatically provided by the TANITA MC-780: average (0–12), high (13–18), and very high (>18).

**Table 2:** Comparison of food consumption between physical activity level groups.

Consumption of Food and Drink	Physical Activity Level (Median with IQR)	Kruskal–Wallis *p*-Value (Low vs. Moderate vs. High)	Effect Size (ε^2^)
Milk and milk product	Low 6.00 (4.00–8.00)	0.973	0.005
	Moderate 5.00 (4.00–7.00)		
	High 6.00 (4.00–8.00)		
Meat, poultry, fish, eggs and products	Low 5.00 (3.00–8.00)	0.191	0.002
	Moderate 6.00 (3.00–8.00)		
	High 5.00 (3.00–8.00)		
Fats, oils and products	Low 3.00 (1.75–5.00)	0.549	0.002
	Moderate 3.00 (2.00–5.00)		
	High 3.5 (2.00–5.00)		
Legumes and cereals	Low 9.00 (5.00–12.00)	0.398	0.003
	Moderate 8.00 (5.00–11.00)		
	High 8.5 (5.00–12.8)		
Sugar, honey and sweets	Low 5.00 (3.00–8.00)	0.782	0.001
	Moderate 5.00 (4.00–8.00)		
	High 5.00 (3.00–9.75)		
Vegetable	Low 14.00 (8.00–23.00)	0.246	0.010
	Moderate 13.00 (7.00–17.00)		
	High 13.00 (7.00–20.80)		
Fruit	Low 4 (2.00–10.30)	0.124	0.001
	Moderate 4.00 (2.00–8.00)		
	High 3.5 (2.00–10.50)		
Snacks and cakes	Low 2.00 (1.00–4.00)	0.178	0.007
	Moderate 2.00 (0.00–4.00)		
	High 1.50 (0.00–4.00)		
Drinks	Low 7.00 (6.00–9.00)	0.095	0.002
	Moderate 8.00 (6.00–9.00)		
	High 7.00 (5.00–9.00)		

The Kruskal–Wallis test was used to compare food-consumption scores across physical activity levels. *p* < 0.05 was considered statistically significant. Effect size was measured by ε^2^. Data are expressed as median with IQR for continuous variables.

**Table 3:** Correlation between food consumption and composition parameters.

Consumption of Food and Drink	Body Composition	Spearman Correlation Coefficient	*p*-Value
Milk and milk product	Body Fat	−0.043	0.562
	Muscle mass	0.153 *	0.037
	Bone mass	−0.007	0.927
	Total body water	−0.033	0.654
	Metabolic age	−0.065	0.376
Meat, poultry, fish, eggs and products	Body Fat	0.091	0.215
	Muscle mass	0.158 *	0.031
	Bone mass	−0.051	0.486
	Total body water	−0.050	0.501
	Metabolic age	−0.108	0.140
Fats, oils and products	Body Fat	0.101	0.168
	Muscle mass	0.120 *	0.101
	Bone mass	−0.059	0.419
	Total body water	−0.056	0.449
	Metabolic age	−0.065	0.376
Legumes and cereals	Body Fat	0.122	0.096
	Muscle mass	0.083	0.260
	Bone mass	0.024	0.742
	Total body water	0.027	0.713
	Metabolic age	0.062	0.403
Sugar, honey and sweets	Body Fat	0.207 *	0.044
	Muscle mass	0.088	0.232
	Bone mass	0.044	0.546
	Total body water	0.030	0.685
	Metabolic age	0.026	0.719
Vegetable	Body Fat	0.143	0.051
	Muscle mass	0.065	0.374
	Bone mass	0.047	0.523
	Total body water	0.054	0.460
	Metabolic age	0.098	0.182
Fruit	Body Fat	0.100	0.173
	Muscle mass	0.141	0.054
	Bone mass	0.047	0.526
	Total body water	0.077	0.296
	Metabolic age	0.088	0.230
Snacks and cakes	Body Fat	0.112	0.731
	Muscle mass	0.104	0.935
	Bone mass	0.126	0.198
	Total body water	0.152	0.211
	Metabolic age	0.109	0.607
Drinks	Body Fat	−0.025	0.128
	Muscle mass	0.006	0.157
	Bone mass	0.095	0.086
	Total body water	0.152 *	0.037
	Metabolic age	0.038	0.139

Spearman’s rank correlation was used for associations between body composition and food-consumption scores; * *p* < 0.05 was considered statistically significant.

**Table 4:** Effect of physical activity level on body composition.

Body Composition	Physical Activity Level (Median with IQR)	Kruskal–Wallis *p*-Value (Low vs. Moderate vs. High)	Effect Size (ε^2^)
Body Fat	Low 0.450 (0.310–0.540)	0.230	0.016
	Moderate 0.410 (0.270–0.550)		
	High 0.380 (0.255–0.477)		
Muscle mass	Low 0.410 (0.340–0.450)	0.564	0.006
	Moderate 0.410 (0.350–0.460)		
	High 0.410 (0.313–0.448)		
Bone mass	Low 2.50 (2.30–3.10)	0.272	0.014
	Moderate 2.60 (2.30–2.90)		
	High 2.45 (2.20–3.05)		
Total body water	Low 33.5 (30.5–42.0)	0.257	0.005
	Moderate 33.1 (30.5–38.6)		
	High 32.1 (29.2–37.0)		
Metabolic age	Low 17.0 (12.0–29.0)	0.645	0.015
	Moderate 16.0 (12.0–26.0)		
	High 14.5 (12.0–20.8)		

Kruskal–Wallis test was used to compare body composition scores across physical activity levels. Data are expressed as median with IQR for continuous variables. *p* < 0.05 was considered statistically significant. Effect size was measured by ε^2^.

## Data Availability

The data used for the present study will be accessible via rational request from the corresponding author responsible.
